# Isolation, identification, and potentiality of gut-derived probiotic bacteria from *Heteropneustes fossilis*, stinging catfish

**DOI:** 10.5455/javar.2024.k806

**Published:** 2024-09-29

**Authors:** Abdul Kader Jilani, Md. Nurul Haider, Abir Hasan, Md. Adil Mahfuz, Md. Nazmul Islam Rifat, Md. Mubarack Hossain, Muhammad Mehedi Hasan

**Affiliations:** Department of Fisheries Technology, Faculty of Fisheries, Bangladesh Agricultural University, Mymensingh, Bangladesh

**Keywords:** Probiotic bacteria, Stinging Catfish, Gut microbiota, pH tolerance, Bile tolerance

## Abstract

**Objective:**

This study was conducted to isolate and identify probiotic bacteria from wild stinging catfish (*Heteropneustes fossilis*), a very popular high-valued aquaculture species of Bangladesh. The isolates were identified through conventional culture-based and molecular techniques.

**Materials and Methods:**

Stinging catfish harvested from natural sources of three sampling sites under two districts (Kishoreganj and Netrakona) were collected, dissected for gut content, and cultured onto *Lactobacillus* MRS Agar plates. Out of 60 bacterial isolates obtained, 10 were chosen for an in vitro evaluation of their probiotic potentials through pH and bile tolerance tests. The 16S rRNA gene sequences of the selected isolates were searched against the NCBI database using the Basic Local Alignment Search Tool for Nucleotides (BLASTNs).

**Results:**

The isolates were identified as *Staphylococcus arlettae*, *Bacillus subtilis*, *Staphylococcus succinus*, *Bacillus velezensis*, *Kocuria subflava*, *Macrococcus caseolyticus*, *Lysinibacillus sphaericus*, *Glutamicibacter mysorens*, *Bacillus cereus*, and *Acinetobacter lwoffii*. Among them, *B. subtilis*, *S. succinus*, *B. velezensis*, *M. caseolyticus*, *G. mysorens*, and *B. cereus* exhibited notable growth across all tested pH levels (pH 2, 3, and 4) and bile salt concentrations (0.3%, 0.5%, and 1.0%) suggesting that they have strong potential as probiotic bacteria. In addition, *S. arlettae *also indicated promising growth except at pH 2.* L. sphaericus and K. subflava *exhibited limited growth at low pH but tolerated bile salt concentrations. *A. lwoffii *did not show any growth at pH tests but minimal growth at the lower concentrations of bile salts.

**Conclusion:**

According to the potentiality assessments and previous literature reviews, five isolates such as *B. subtilis, S. succinus, M. caseolyticus, G. mysorens*, and *B. cereus* were identified as potential probiotic bacteria. As species-specific probiotics are considered to perform more effectively and efficiently than unknown-sourced commercial probiotics, the findings of this study will be applicable in enhancing the aquaculture production of stinging catfish.

## Introduction

The concept of probiotic (Greek “*pro bios*” means for life) microorganisms was introduced in 1907 by Russian zoologist, Elie Metchnikoff. Probiotics are “live microorganisms which when administered in adequate amounts confer a health benefit on the host” [1]. Various Gram-positive (*Bacillus, Carnobacterium, Enterococcus, Lactobacillus, Lactococcus, Micrococcus, *and* Streptococcus*) and Gram-negative bacteria (*Aeromonas, Alteromonas, Photorhodobacterium, Pseudomonas, *and* Vibrio*) are identified as probiotics [2]. Most isolated probiotics that produce lactic acid help prevent the proliferation of harmful Gram-positive or Gram-negative bacteria [3].

Probiotics benefit the host by enhancing health, promoting growth performance, improving stress tolerance, optimizing feed utilization, and increasing disease resistance. They also help to reduce environmental impact by decreasing ammonia production and waste in aquatic systems [4]. They benefit fish by reducing the quantities of nitrite, nitrate, and ammonium. Additionally, they strengthen immune systems, promote illness resistance, and resist harmful infections. Sometimes, they even promote larval growth without antibiotics [3]. Probiotics also showed inhibitory activity against certain harmful bacteria like *Aeromonas hydrophila, *and* Aeromonas salmonicida *and antibiotic-resistant activity against some antibiotics such as streptomycin, vancomycin, and so on [5]. They can generate an immune response either at the cellular or humoral level and help the host by engaging in competitive exclusion, competing with potential pathogens for resources like oxygen, nutrients, space, and so on [2]. Probiotics enhance feed utilization by increasing digestive enzyme activity and breaking down anti-nutritional compounds in feedstuffs, thereby reducing overall production costs [6].

Annual economic losses in the fish culture industry around the world have been reported due to outbreaks of bacterial disease. The use of antibiotics in disease treatment led to the rise of natural antibiotic resistance. That is why public health organizations confined the use of antibiotics and recommended the development of new techniques for disease treatment. Therefore, biological disease control has received widespread attention in the last decade. An alternative and effective approach to antibiotic administration in aquaculture is probiotics [7]. Since the intestinal microbial community of fishes is poorly understood and consists of species-specific bacteria, it is critical to separate and recognize probiotic bacteria particular to each species [8].

The *Heteropneustes fossilis* (Bloch), stinging catfish, a highly valued popular aquaculture fish species was chosen for isolating and identifying gut probiotic bacteria. This fish is commercially important and also popular among the consumers of Bangladesh because of its nutritional and therapeutic benefits. Compared to many other freshwater fishes, it has a high iron level (226 mg/100 gm) and relatively high calcium content. Because it is a lean fish, it is ideal for persons who are allergic to animal fats. However, over-exploitation and ecological changes have led to its decline. Despite its high market value and consumer demand, this fish species has enormous aquaculture potential [9]. Several commercially available imported probiotics have been using commonly in the culture system of stinging catfish without considering their efficiencies and effectiveness. The present study was conducted to isolate and identify gut probiotic bacteria (lactic acid bacteria) from wild stinging catfish. The selected isolates were also detected through conventional culture-based and molecular techniques (16S rRNA gene sequencing). The potentiality of the isolates as probiotics was also tested through their pH and bile tolerance attributes.

## Materials and Method

### Ethical statement

The research protocol was approved and the research was performed according to the guidelines of the Animal Welfare and Experimentation Ethics Committee (AWEEC) of Bangladesh Agriculture University in Mymensingh [AWEEC/BAU/2024 (33)].

### Collection of gut samples from the wild stinging catfish

**Figure 1.**
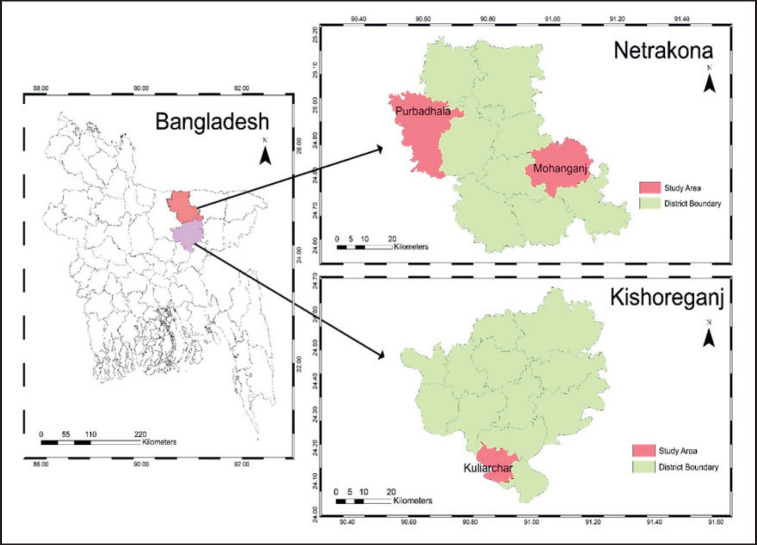
Experimental sites and locations considered for the collection of wild stinging catfish for the isolation and identification of probiotic bacteria.

The stinging catfish specimens were collected from three stations namely, Kuliarchar upazila in Kishoreganj (Station 1), Purbadhala upazila (Station 2), and Mohanganj upazila in Netrokona (Station 3), Bangladesh (Fig. 1). The collected freshwater fish were cleaned using sterile distilled water [5]. Then, the fish were dissected from the rectum towards the head under sterilized conditions to remove the digestive systems, aiming to isolate probiotic bacteria. The gut content was completely ground with sterilized scissors.

### Isolation, cultivation, and characterization of gut-derived bacterial strains

A selective *Lactobacillus* MRS (De Man, Rogosa, and Sharpe) agar media was used for the isolation and cultivation of lactic acid-producing bacteria. The low pH of this medium, which is tolerated by probiotic gut bacteria, inhibits the development of other dominating organisms in human feces [10]. The collected gut samples were then inoculated in freshly prepared MRS agar plates by swabbing and streaking methods with the help of sterilized cotton. The gut samples were also inoculated in MRS and nutrient broth media for further experiment. The plates and broth were then incubated at 37°C temperature aerobically in an incubator for 24–48 h to obtain isolated colonies [10,11].

Based on the similarities in colony characteristics (such as size, shape, pigments, and colony margins), as representative 10 isolates were selected for molecular identification and biochemical (pH and bile salt) tolerance tests.

### Molecular identification of probiotic bacteria

Molecular identification of probiotic bacteria involves extracting DNA from samples, amplifying the 16S rRNA gene through PCR using universal primers, and performing gel electrophoresis. The amplified DNA was then sequenced using a Sanger sequencer, followed by data analyses. The details of the protocol are described as follows.

### Bacterial genomic DNA extraction and PCR amplification protocol

The phenol: chloroform extraction method was used for DNA extraction described by Wright et al. [12] with slight modification. A set of universal primers, 27F (5´-AGA GTT TGA TCC TGG CTC AG-3´) and 1492R (5´-ACG GYT ACC TTG TTA CGA CTT-3´), was used to amplify the 1400 bp region of the 16S rRNA gene. The polymerase chain reaction (PCR) was conducted in a 25 μl reaction volume, which included 10 ng of template DNA, 2 mM MgCl2, 1 μl of each primer (10 mM), and 1 × *Taq* Master Mix. The PCR conditions were as follows: initial denaturation at 95°C for 5 min, followed by 35 cycles of denaturation at 94°C for 30 sec, annealing at 54°C for 30 sec, and extension at 72°C for 45 sec, with a final extension at 72°C for 10 min. The gel documentation system (Bio-Rad) was used to visualize the products. A 1 Kb plus DNA ladder (New England Biolabs, UK) was used to compare the bands on an agarose gel. The PCR products were verified using 1.5% agarose gels stained with ethidium bromide and examined with the EZEE Clearview UV transilluminator.

### Sequencing protocol

PCR products were purified, and then single-stranded products were generated using cycle sequencing PCR with forward or reverse primers. PCR products were run on a Sanger sequencing machine using the dideoxy chain termination method at Wuhan Tianyi Huayu Gene Technology Co., Ltd., according to the manufacturer’s protocol.

### Analyses of the sequence data

The sequence data were compared to GenBank entries (https://www.ncbi.nlm.nih.gov/) using the Basic Local Alignment Search Tool for Nucleotides (BLASTNs) available on the NCBI website to identify the isolates. Multiple sequence alignment was carried out with the CLUSTALW program. Phylogenetic analysis was performed using “Mega 11” software, employing the neighbor-joining method to construct the phylogenetic tree.

### Biochemical potentiality tests

The pH and bile tolerance tests are crucial for assessing probiotic bacterial ability to survive and act in the harsh conditions of the gastrointestinal tract of organisms. Probiotic bacteria must be resistant to the acidity of the stomach, bile, and pancreatic enzymes in the alimentary canal [13,14].

### pH tolerance test

To perform the pH tolerance test, bacterial isolates were first grown on MRS agar media and then suspended in sterile physiological saline (0.9% sodium chloride solution). Three pH solutions were prepared using hydrochloric acid or sodium hydroxide and adjusted the levels at pH 2.0, pH 3.0, and pH 4.0. The bacterial suspension was then mixed with corresponding pH solutions. For the viability of the bacterial strains, the subsamples were taken and plated onto *Lactobacillus* MRS agar plates at various time intervals such as 0, 30, 60, and 90 min of treatment [5,13,14]. As a control, only the bacterial cells suspended in the physiological saline were plated similarly onto the *Lactobacillus* MRS agar plates without any pH treatment. The plates were then incubated at 37°C, growth and colony formation were observed, and colonies were counted and calculated using the following formula:

Viable count of bacteria (CFU/ml) = (No. of colonies on agar plate × total dilution factor)/volume of culture plated in ml.

### Bile salt tolerance test

In the bile salt tolerance test, similarly bacterial isolates were grown on *Lactobacillus* MRS agar media and then suspended in sterile physiological saline (0.9% sodium chloride solution). Various bile salt solutions of concentrations 0.3%, 0.5%, and 1.0% at pH 8.0 were prepared. Bacterial suspensions were then mixed with the corresponding bile solutions, subsamples were taken and plated onto *Lactobacillus* MRS agar plates at various time intervals (after 0, 30, 90, and 180 min of treatment) for the viability assessment [5,13,14]. Bacterial strains suspended to the physiological saline (without any bile salt treatment) were plated onto the *Lactobacillus* MRS agar plates at similar time intervals and considered as controls. All the plates were then incubated at 37°C, and the colonies were counted and calculated using the following formula:

Viable count of bacteria (CFU/ml) = (No. of colonies on agar plate × total dilution factor) /volume of culture plated in ml.

### Statistical analysis

The data were collected and recorded using MS Excel 2013. The results were then evaluated based on data obtained in triplicate and presented in table formats. The data were analyzed using SPSS software, with statistical significance set at *p* < 0.05.

## Results

### Isolated gut bacteria from wild stinging catfish

From the 3 sampling stations, 60 bacterial isolates were obtained from the gut content cultures of the stinging catfish. Among them, 20 isolates were obtained from station 1 (from Kuliarchar upazila), 19 isolates from station 2 (from Purbadhala upazila), and 21 isolates from station 3 (from Mohanganj upazila). These isolates were eventually temporarily preserved in agar slants for further examination, identification, and tests.

### Selection of bacterial isolates for molecular detection

Finally, 10 isolates were chosen (based on the similarities and dissimilarities of their colony characteristics) for molecular identification and further potentiality tests through pH and bile tolerance. Among them, 3 were selected from station 1, 2 from the station 2, and 5 from the station 3.

### Identification of the isolates through molecular detection

**Figure 2.**
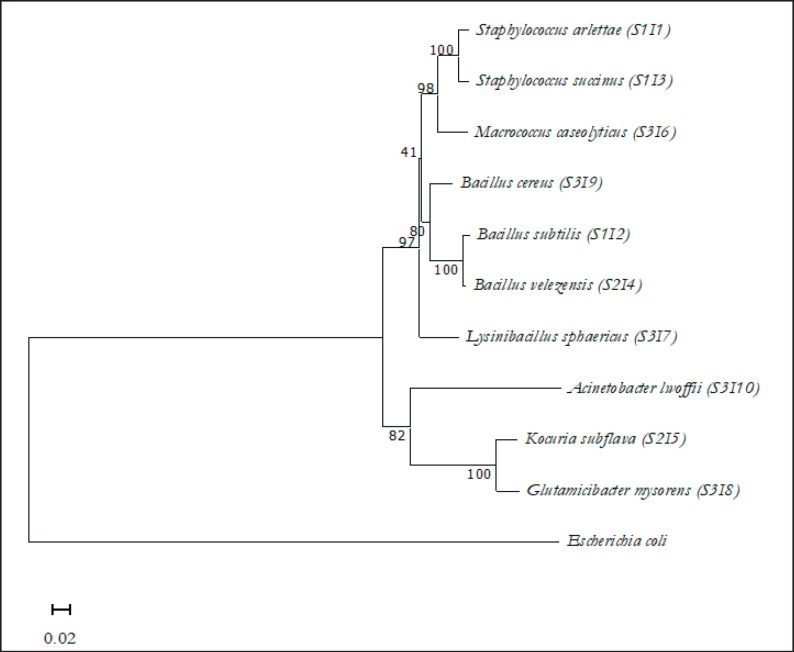
Dendrogram showing phylogenetic relations and evolutionary relationships among the isolates. Horizontal bars in the dendrogram represent the branch length and the bootstrap values represent the similarity and homology of the neighboring sequences. The scale bar represents 0.02 substitutions for each position of a nucleotide. *Escherichia coli* represents an out-group. The letters S and I indicate the station (S) and isolate number (I), respectively.

The 16S rRNA of the isolates were sequenced using Sanger for their molecular identification. Sequence analysis was carried out using Bioedit software. To assess the similarity of the obtained sequences, the NCBI (National Center for Biotechnology Information) database was utilized as standard sequence data. In terms of the percentage of similarity detected, it ranges from 98.97% to 100%. The sequence with the highest degree of similarity to a specific species is considered the expected species. After analysis, they were identified as, *Staphylococcus arlettae* (S1I1)*, Bacillus subtilis* (S1I2)*, Staphylococcus succinus* (S1I3)*, Bacillus velezensis* (S2I4)*, Kocuria subflava *(S2I5)*, Macrococcus caseolyticus *(S3I6)*, Lysinibacillus sphaericus *(S3I7)*, Glutamicibacter mysorens *(S3I8)*, Bacillus cereus* (S3I9)*, and Acinetobacter lwoffii *(S3I10) (Table 1). The phylogenetic and evolutionary relationships among the identified isolates were presented in the dendrogram/phylogenetic tree (Fig. 2). In the figure, the scale bar represents 0.02 substitutions for each position of a nucleotide and *Escherichia coli* represents an out-group.

### pH tolerance tests of the selected isolates

The differences in bacterial growth patterns between the control (no pH treatment; cells suspended in the physiological saline were plated only) and at pH levels 2, 3, and 4 after 90 min of treatment were tested through a 2-tailed Pearson Correlation test (Table 2). Compared to the control condition, the highest bacterial survival rate after 90 min of pH treatment was observed at pH 4 (7 isolates out of 10 showed growth). The Pearson correlation analysis revealed a significant difference in survival between pH 2 and the control condition (*p* < 0.05; 2-tailed Pearson Correlation test; Table 2).

The dynamic growth response was evaluated after various pH values. Some isolates had shown excellent growth patterns in every pH concentration and time, while others showed moderate to no response (Table 3). After treatment at pH 2, *Staphylococcus arlettae* (S_1_I_1_)*,* *Kocuria subflava *(S_2_I_5_)*,* *Lysinibacillus sphaericus *(S_3_I_7_)*,* and* Acinetobacter lwoffii *(S_3_I_10_) did not grow at all, while other isolates had shown some growth patterns. For example, *Bacillus subtilis* (S1I2)*, Bacillus velezensis* (S_2_I_4_)*, *and *Bacillus cereus* (S_3_I_9_) exhibited significant growth (3.55×10^4^ CFU/ml to 1.77×10^4^ CFU/ml) at all treatment periods (0, 30, 60, and 90 min of treatment), with a reduction in cell counts (1.55×10^4^ CFU/ml) when the treatment period was increased. The remaining isolates were mediocre and showed no growth at 90 min of treatment time. Logically, relatively higher survival of the isolates was observed in the case of pH 4 treatments (4.44×10^4^ CFU/ml to 1.11×10^4^ CFU/ml), where only *Acinetobacter lwoffii *(S_3_I_10_) did not show any growth. *Staphylococcus arlettae* (S_1_I_1_)*, Bacillus subtilis* (S_1_I_2_)*, Staphylococcus succinus* (S_1_I_3_)*, Bacillus velezensis* (S_2_I_4_)*, Macrococcus caseolyticus *(S_3_I_6_)*, Glutamicibacter mysorens *(S_3_I_8_)*, *and *Bacillus cereus* (S3I9) were able to grow at every treatment period whereas, *Kocuria subflava *(S_2_I_5_) and *Lysinibacillus sphaericus *(S3I7) showed survivability only at 0 min of treatment (1.99×10^4^ CFU/ml and 1.66×10^4^ CFU/ml, respectively) (Table 3).

**Table 1.** Molecular identification of the selected isolates through 16S rRNA sequencing using the Sanger method. The identified isolates represent potential probiotic strains from the gut of *H. fossilis*.

Isolate IDs	Species identified	Percent of similarity identified	Primer used in identification	Source	Methodology
S_1_I_1_*	*Staphylococcus arlettae*	98.97	27F and 1492R	National Center for Biotechnology Information (NCBI) database	Using n-BLAST
S_1_I_2_	*Bacillus subtilis*	100			
S_1_I_3_	*Staphylococcus succinus*	99.76			
S_2_I_4_	*Bacillus velezensis*	99.87			
S_2_I_5_	*Kocuria subflava*	100			
S_3_I_6_	*Macrococcus caseolyticus*	100			
S_3_I_7_	*Lysinibacillus sphaericus*	99.37			
S_3_I_8_	*Glutamicibacter mysorens*	99.88			
S_3_I_9_	*Bacillus cereus*	99.76			
S_3_I_10_	*Acinetobacter lwoffii*	100			

*the letters S, and I in the isolate IDs indicate, S = station, and I =isolate number

**Table 2.** Statistical analysis of bacterial growth at pH levels 2, 3, and 4 compared to the control after 90 minutes of treatment using the Pearson correlation method to assess similarities and differences.

		Bacterial growth in the control condition	Bacterial growth after 90 min of treatment in pH 2	Bacterial growth after 90 min of treatment in pH 3	Bacterial growth after 90 min of treatment in pH 4
Bacterial growth in the control condition	Pearson Correlation	1.0	0.436	0.775**	0.846**
	Sig. (2-tailed)		0.208	0.009	0.002
	N	10	10	10	10
Bacterial growth after 90 min of treatment in pH 2	Pearson Correlation	0.436	1.0	0.680*	0.728*
	Sig. (2-tailed)	0.208		0.031	0.017
	N	10	10	10	10
Bacterial growth after 90 min of treatment in pH 3	Pearson Correlation	0.775**	0.680*	1.0	0.810**
	Sig. (2-tailed)	0.009	0.031		0.005
	N	10	10	10	10
Bacterial growth after 90 min of treatment in pH 4	Pearson Correlation	0.846**	0.728*	0.810**	1.0
	Sig. (2-tailed)	0.002	0.017	0.005	
	N	10	10	10	10
**. Correlation is significant at the 0.01 level (2-tailed).					
*. Correlation is significant at the 0.05 level (2-tailed).					

### Bile salt tolerance tests of the selected isolates

The differences in bacterial growth patterns between the control (no bile salt treatment; cells suspended in the physiological saline were plated only) and at different bile salt concentrations (1%, 0.5%, 0.3%) after 180 min of treatment were tested though 2-tailed Pearson Correlation test (Table 4). Compared to the control condition, the highest bacterial survival rate after 180 min of bile treatment was observed at a 0.3% concentration (9 isolates out of 10 showed growth). The Pearson correlation analysis showed no significant differences in survival at 1%, 0.5%, and 0.3% bile salt concentrations compared to the control condition (*p *< 0.05; 2-tailed Pearson correlation test; Table 4).

The growth response varied at various bile salt concentrations. Unlike pH, almost all the isolates showed growth after treatments in every concentration (1%, 0.5%, 0.3%) of bile salt, however, less growth was observed when the concentrations were increased (Table 5). Among the isolates, only *Acinetobacter lwoffii *(S_3_I_10_) did not show any growth response after 1% bile salt treatment. While, *Bacillus subtilis* (S_1_I_2_)*, Staphylococcus succinus* (S_1_I_3_)*, Bacillus velezensis* (S_2_I_4_)*, Macrococcus caseolyticus *(S_3_I_6_)*, Glutamicibacter mysorens *(S3I8)*,* and* Bacillus cereus* (S3I9) were proliferated (3.77×10^4^ CFU/ml to 1.77×10^4^ CFU/ml) at every treatment period (0, 30, 90, and 180 min of treatment) regardless of the concentrations. Almost all the isolates had shown a relatively higher growth (3.99×10^4^ CFU/ml to 0.99×10^4^ CFU/ml) after 0.3% bile salt treatment at every interval (0, 30, 90, and 180 min of treatment); however, *Acinetobacter lwoffii *(S_3_I_10_) did not grow after 180 min-treatment time (Table 5).

## Discussion

Fish have very complex microbial ecosystems in their gastrointestinal tracts, which are crucial for host immunity and nutrition. Evaluating the microbiota of the gastrointestinal tracts of fish helps to identify potential probiotics and harmful bacteria, which will enhance our understanding regarding the microbial nature of the fish gut and health management of the catfish [15,16]. In Bangladesh, probiotics are used to some extent, but the ratio of use is still low. In the Mymensingh region, only 8.33% of farms use gut probiotics [17]. Due to its impact on host nutrition, immunological state, disease susceptibility, growth, and reproduction, the gut microbiome has attracted a lot of research recently. The wild individuals were selected because they are likely to withstand the harshest environment for survival. For that reason, they are the strongest one and their gut microbiota may be effective for the culture of that species.

**Table 3.** Changes in viable counts of the isolates (mean×10^4^ CFU/ml) after treatment with different pH concentrations and periods (including standard deviations). The isolates were obtained from the gut of *Heteropneustes fossilis* to identify and test their potentiality as probiotics.

Isolate IDs	Control* (mean ± SD)	pH-2				pH-3				pH-4			
		Treatment period (min)				Treatment period (min)				Treatment period (min)			
		0	30	60	90	0	30	60	90	0	30	60	90
		(Mean ± SD)	(Mean ± SD)	(Mean ± SD)	(Mean ± SD)	(Mean ± SD)	(Mean ± SD)	(Mean ± SD)	(Mean ± SD)	(Mean ± SD)	(Mean ± SD)	(Mean ± SD)	(Mean ± SD)
S_1_I_1_**	3.66 ± 0.81	NG***	NG	NG	NG	2.88 ± 0.47	3.22 ± 0.77	1.66 ± 0.53	NG	3.44 ± 0.77	3.11 ± 0.47	2.77 ± 0.47	1.88 ± 0.88
S_1_I_2_	4.33 ± 0.30	3.11 ± 0.17	3.22 ± 0.47	2.88 ± 0.17	1.99 ± 0.30	3.77 ± 0.47	3.11 ± 0.61	2.77 ± 0.47	1.66 ± 0.17	4.44 ± 0.99	3.33 ± 0.30	2.99 ± 0.61	2.55 ± 0.47
S_1_I_3_	4.44 ± 0.47	3.11 ± 0.77	1.55 ± 0.64	NG	NG	3.88 ± 0.47	3.33 ± 0.35	1.77 ± 0.47	1.22 ± 0.35	4.22 ± 1.0	3.11 ± 0.77	2.33 ± 0.53	1.55 ± 0.17
S_2_I_4_	4.44 ± 0.64	3.55 ± 0.64	2.88 ± 0.64	2.22 ± 0.30	1.77 ± 0.47	3.11 ± 0.47	2.88 ± 0.30	2.44 ± 0.47	2.22 ± 0.17	3.22 ± 0.17	3.66 ± 0.81	2.88 ± 0.30	2.66 ± 0.81
S_2_I_5_	1.99 ± 0.30	NG	NG	NG	NG	NG	NG	NG	NG	1.99 ± 0.53	NG	NG	NG
S_3_I_6_	3.88 ± 0.17	3.22 ± 0.64	3.11 ± 0.64	2.11 ± 0.47	NG	3.55 ± 0.64	3.11 ± 0.47	2.66 ± 1.0	1.33 ± 0.30	3.99 ± 0.81	2.88 ± 0.64	2.66 ± 0.53	1.11 ± 0.17
S_3_I_7_	2.88 ± 0.47	NG	NG	NG	NG	NG	NG	NG	NG	1.66 ± 0.61	NG	NG	NG
S_3_I_8_	4.22 ± 0.35	3.55 ± 0.88	1.99 ± 0.53	NG	NG	4.22 ± 0.64	3.33 ± 0.30	2.11 ± 0.77	1.77 ± 0.47	3.77 ± 0.47	3.55 ± 0.47	2.44 ± 0.64	1.77 ± 0.17
S_3_I_9_	3.66 ± 0.47	3.11 ± 0.30	2.88 ± 0.47	2.44 ± 0.30	1.55 ± 0.17	4.22 ± 0.81	3.88 ± 0.17	2.88 ± 0.30	1.88 ± 0.35	3.77 ± 0.47	2.99 ± 0.61	3.11 ± 0.81	2.22 ± 0.47
S_3_I_10_	1.33 ± 0.30	NG	NG	NG	NG	NG	NG	NG	NG	NG	NG	NG	NG
* No pH treatment; cells suspended in the physiological saline were plated only. ** The letters S, and I in the isolate IDs indicate, S = station, and I =isolate number. *** NG = No growth.													
Lower					Higher								

**Table 4.** Statistical analysis of bacterial growth at 1%, 0.5%, and 0.3% bile concentrations compared to the control after 180 min of treatment using the Pearson correlation method to assess similarities and differences.

		Bacterial growth in the control condition	Bacterial growth after 180 min of treatment in 1% bile concentration	Bacterial growth after 180 min of treatment in 0.5% bile concentration	Bacterial growth after 180 min of treatment in 0.3% bile concentration
Bacterial growth in the control condition	Pearson Correlation	1.0	0.823**	0.828**	0.725*
	Sig. (2-tailed)		0.003	0.003	0.018
	N	10	10	10	10
Bacterial growth after 180 min of treatment in 1% bile concentration	Pearson Correlation	0.823**	1.0	0.707*	0.782**
	Sig. (2-tailed)	0.003		0.022	0.007
	N	10	10	10	10
Bacterial growth after 180 min of treatment in 0.5% bile concentration	Pearson Correlation	0.828**	0.707*	1.0	0.586
	Sig. (2-tailed)	0.003	0.022		0.075
	N	10	10	10	10
Bacterial growth after 180 min of treatment in 0.3% bile concentration	Pearson Correlation	0.725*	0.782**	0.586	1.0
	Sig. (2-tailed)	0.018	0.007	0.075	
	N	10	10	10	10
**. Correlation is significant at the 0.01 level (2-tailed).					
*. Correlation is significant at the 0.05 level (2-tailed).					

Maximum diversification was achieved by selecting three upazilas, with each one representing a station, from which a total of 60 isolates were obtained and 10 were selected, ensuring representation from each station. Though the isolation of probiotics from the gut of stinging catfish has not been well-established, the isolation of gut bacteria from other catfish and freshwater fish has been successfully conducted. Such as the results found in the stomach of yellow catfish revealed a variety of bacteria, including species from the genera *Bradyrhizobium*, *Hafnia*, *Plesiomonas*, *Pseudomonas*, *Phyllobacterium,* and *Bacillus*, which were retrieved from the gastrointestinal contents [15].

To identify the selected gut bacteria, 16s rRNA gene sequencing was applied by the researcher, and different probiotic bacteria such as *Bacillus cereus, Staphylococcus*, *Arthrobacter*, *Rhodococcus, Acinetobacter, Propionibacterium*, and *Streptococcus* were identified. Using the same 16S rRNA sequence, various gut bacterial isolates were identified. These isolates differ in their source in nature and their functional properties. The distinctive source and functional properties of these identified isolates are summarized in (Table 6).

Based on *in vitro* selection (pH and bile tolerance tests), among the identified isolates, most of them were found to have probiotic characteristics. *Staphylococcus arlettae* (S_1_I_1_) was discovered in the digestive systems of fish and shellfish, and it was found to possess antimicrobial activity [18]. It has probiotic potential, and it can withstand low pH and various bile salt concentrations. In the present study, this strain showed survival and proliferation up to pH 3 and significant growth at pH 4 (Table 3). Moreover, it showed a growth pattern as high as 1% of bile salt concentration with significant growth at 0.5% and 0.3% concentrations (Table 5). From the same genus, *Staphylococcus succinus* (S_1_I_3_) exhibited antagonistic and proteolytic activity. It is also considered a beneficial bacterium in aquaculture and can resist the growth of *Escherichia coli* and can withstand low pH and high bile salt concentrations which were up to 1%. This strain could be used as a biocontrol agent in different fields of research [19]. In the present study, the same survival pattern was found which is significant growth in pH 3, pH 4, and up to 1% bile salt concentrations (Table 3). Regarding the analyses, these strains from the *Staphylococcus* genus can be accounted for as a probiotic bacterium for our selected fish species.

**Table 5.** Changes in viable counts of the isolates (mean×10^4^ CFU/ml) after treatment with different bile salt concentrations and periods (including standard deviations). The isolates were obtained from the gut of *Heteropneustes fossilis* to identify and test their potentiality as probiotics.

Isolate IDs	Control* (Mean ± SD)	1% Concentration				0.5% Concentration				0.3% Concentration			
		Treatment period (min)				Treatment period (min)				Treatment period (min)			
		0	30	90	180	0	30	90	180	0	30	90	180
		(Mean ± SD)	(Mean ± SD)	(Mean ± SD)	(Mean ± SD)	(Mean ± SD)	(Mean ± SD)	(Mean ± SD)	(Mean ± SD)	(Mean ± SD)	(Mean ± SD)	(Mean ± SD)	(Mean ± SD)
S_1_I_1_**	3.33 ± 0.61	2.22 ± 0.17	1.66 ± 0.30	0.99 ± 0.30	NG***	2.44 ± 0.47	1.88 ± 0.17	1.22 ± 0.35	1.33 ± 0.30	3.55 ± 0.17	2.88 ± 0.17	2.11 ± 0.17	1.66 ± 0.30
S_1_I_2_	3.99 ± 0.30	3.22 ± 0.47	2.77 ± 0.17	1.55 ± 0.30	0.88 ± 0.47	3.33 ± 0.35	3.22 ± 0.61	2.22 ± 0.47	1.77 ± 0.17	3.77 ± 0.61	3.22 ± 0.30	2.55 ± 0.17	1.99 ± 0.30
S_1_I_3_	3.77 ± 0.17	3.77 ± 0.35	2.77 ± 0.17	2.11 ± 0.47	1.44 ± 0.17	3.55 ± 0.35	3.11 ± 0.35	2.55 ± 0.47	1.44 ± 0.47	4.77 ± 0.35	3.99 ± 0.30	3.22 ± 0.47	2.88 ± 0.17
S_2_I_4_	3.88 ± 0.30	2.88 ± 0.35	2.77 ± 0.17	1.99 ± 0.30	1.22 ± 0.35	3.11 ± 0.47	3.44 ± 0.77	2.99 ± 0.64	2.22 ± 0.17	3.66 ± 0.17	3.22 ± 0.47	2.88 ± 0.30	2.55 ± 0.17
S_2_I_5_	1.99 ± 0.30	1.55 ± 0.35	1.11 ± 0.17	NG	NG	1.99 ± 0.30	1.55 ± 0.17	1.22 ± 0.35	NG	2.88 ± 0.47	3.44 ± 0.47	2.55 ± 0.17	1.99 ± 0.30
S_3_I_6_	3.33 ± 0.61	2.88 ± 0.35	2.66 ± 0.30	1.66 ± 0.30	0.99 ± 0.30	3.22 ± 0.17	3.11 ± 0.17	2.44 ± 0.47	2.11 ± 0.17	3.99 ± 0.30	3.22 ± 0.47	2.77 ± 0.17	1.77 ± 0.17
S_3_I_7_	2.88 ± 0.17	1.88 ± 0.35	1.44 ± 0.17	1.11 ± 0.17	NG	1.88 ± 0.17	1.66 ± 0.30	1.55 ± 0.35	1.22 ± 0.17	3.22 ± 0.47	2.44 ± 0.17	1.55 ± 0.35	0.99 ± 0.30
S_3_I_8_	4.33 ± 0.30	2.88 ± 0.17	1.88 ± 0.35	1.99 ± 0.30	1.33 ± 0.30	3.11 ± 0.17	2.11 ± 0.47	1.77 ± 0.17	1.33 ± 0.30	4.66 ± 0.30	2.88 ± 0.35	4.22 ± 0.17	2.44 ± 0.17
S_3_I_9_	3.88 ± 0.81	2.77 ± 0.35	2.11 ± 0.47	1.66 ± 0.30	1.44 ± 0.35	3.33 ± 0.61	3.11 ± 0.30	2.44 ± 0.17	1.99 ± 0.30	3.99 ± 0.47	3.22 ± 0.35	2.88 ± 0.17	2.55 ± 0.47
S_3_I_10_	1.99 ± 0.30	NG	NG	NG	NG	0.88 ± 0.17	0.77 ± 0.17	NG	NG	1.99 ± 0.30	0.99 ± 0.30	0.77 ± 0.17	NG
* No pH treatment; cells suspended in the physiological saline were plated only. ** The letters S, and I in the isolate IDs indicate, S = station, and I =isolate number. *** NG = No growth.													
Lower					Higher								

**Table 6.** Background information about the identified isolates obtained from the gut of *Heteropneustes fossilis*, detailing their source and functional properties.

Isolate IDs	Species identified	Sources in nature	Metabolism/Functional properties	References
S_1_I_1_*	*Staphylococcus arlettae*	The gut of fish and shellfish	Antimicrobial activity	[18]
S_1_I_2_	*Bacillus subtilis*	Worldwide; e.g.: soil, air, freshwater, and seawater	Metabolism through the breakdown of protein, carbs, and complex lipids, antimicrobial activity	[20]
S_1_I_3_	*Staphylococcus succinus*	Upper gastrointestinal tract	Antagonistic activity against uropathogenic and proteolytic activity	[19]
S_2_I_4_	*Bacillus velezensis*	Wide distribution; e.g.: fish and shrimp intestines, soil, manure, and domestic yak	Synthesization of many ribosomal and non-ribosomal bioactive metabolites, anti-bacterial and anti-fungal activities	[22]
S_2_I_5_	*Kocuria subflava*	Soil, water	Catalase-positive, oxidase-negative	[28]
S_3_I_6_	*Macrococcus caseolyticus*	Cow’s milk, bovine organs and food-processing factories.	Starch metabolism, coagulase-negative and catalase-positive	[29]
S_3_I_7_	*Lysinibacillus sphaericus*	Fish intestine, cow milk, curd, cheese	Carbohydrate fermentation, antimicrobial activity against *E. coli,* and catalase-negative	[26]
S_3_I_8_	*Glutamicibacter mysorens*	Soil, sediment, and intestine of marine and freshwater fish	Antiproliferative activity of antimicrobial peptides and bioactive compounds, catalase-positive	[30]
S_3_I_9_	*Bacillus cereus*	Gastrointestinal tract, soil, air, fermented food	Amino acid, carbohydrate, and lipid metabolism, antimicrobial, antioxidant, and vitamin production	[21]
S_3_I_10_	*Acinetobacter lwoffii*	Soil, water, freshwater, and marine water fish	Saprophytic, catalase-positive and oxidase-negative	[31]

*the letters S, and I in the isolate IDs indicate, S = station, and I =isolate number

Among the family Bacillaceae, 3 strains were obtained from the genus *Bacillus* and 1 from the genus *Lysinibacillus*. These are *B. subtilis *(S_1_I_2_), *B. velezensis *(S_2_I_4_), *B. cereus *(S_3_I_9_), and *L. sphaericus *(S_3_I_7_). *Bacillus subtilis* (S_1_I_2_) was discovered worldwide including in soil, air, and freshwater and it was proven to have antimicrobial activity [20]. The probiotic potential of *B. subtilis* was examined in the Indian major carp and found that the growth rate of the *Labeo rohita* was higher when fed *B. subtilis-*containing feed. It was also observed that the colonization of *B. subtilis* in the gut epithelium reduced the probability of harmful bacterial infection and consequently, fish can develop the ability to protect themselves from numerous diseases [20]. Additionally, *B. subtilis* has antimicrobial and antiviral activity and can inhibit the adhesion of *Salmonella enteritidis*, *Listeria monocytogenes*, and *Escherichia coli *[21]. In the present study, this strain was able to grow at low pH (e.g., up to pH 2) (Table 3) and bile salt concentration as high as 1% (Table 5). Since to become a probiotic organism, it is important to exhibit acid and bile salt tolerance [13,14], this strain can be used as a probiotic organism to reduce the pathogenic attack in the culture of stinging catfish. *Bacillus velezensis* (S_2_I_4_) was also isolated worldwide including fish and shrimp intestine. It is known to produce numerous bioactive metabolites, both ribosomal and non-ribosomal. These include surfactin, fengycin, bacillibactin, difficidin, bacillaene, macrolactin, bacilysin, and acetoin, as well as various volatile organic compounds. It has significant antibacterial and antifungal effects that can easily be isolated and cultured for application in aquaculture [22]. Additionally, this strain showed significant tolerance in a pH range of 2-9 and survived in the presence of bile salt up to 1% concentration [23]. In the present study, the same result was found which showed the growth in every pH for every treatment time (Table 3), and in all bile salt concentrations (Table 5). So, this strain can be considered a good probiotic for our selected fish species.

*Bacillus cereus* (S_3_I_9_) is one of the most important and well-known probiotics from the *Bacillus* genus that is employed in aquaculture and it can produce vitamins and has antibacterial, anticancer, antioxidant, and other characteristics [21]. This strain possesses growth-promoting potential, serves as a probiotic, and positively influences the growth of aquatic organisms [24]. It demonstrated probiotic potential by enhancing growth, immunity, and disease resistance in sea cucumbers, as well as improving growth performance in stinging catfish (*H. fossilis*) [25]. In the present study, this strain showed a very good growth response in all pH, including pH 2 (Table 3), and in high bile salt concentration which was 1% (Table 5). Combining this information, we can consider *B. cereus* to be one of the most important probiotics for stinging catfish. Last but not least from the family Bacillaceae is *Lysinibacillus sphaericus* (S_3_I_7_). This strain exhibited probiotic properties like antimicrobial activity against pathogenic organisms [26] and inhibitory effects against *Vibrio harveyi *and *V. parahaemolyticus* [27]. In the present study, no significant growth was found in low pH with a little response at pH 4 in 0 min of treatment (Table 3). On the contrary, it showed a good response in bile salt concentration while having no growth at 180 min of treatment at 1% concentration (Table 5). So, it can be concluded that this strain may have a moderate impact on our target species.

*Kocuria subflava* (S_2_I_5_) is a novel bacterium, and there is still much unknown about it. It was isolated from the soil and water of the Indian Ocean, and its pH tolerance range was found to be between 5 and 10 [28]. In the present study, no significant growth was found in pH 2 and 3, whereas very little growth was observed at pH 4 in 0 min of treatment (Table 3). Compared to that, this strain showed a good response in 0.3% bile concentration, a little less in 0.5%, and low in 1% concentration (Table 5). As it is a novel bacterium, further research is needed to understand its probiotic characteristics. *Macrococcus caseolyticus* (S_3_I_6_) is closely linked to *Bacillus* species in addition to *Staphylococcus* species, and this strain contains genes for both amylase and glycogen production, which are highly similar to those found in *Bacillus* species [29]. In the present study, the growth response in pH 3 and 4 was significant. On the other hand, in pH 2, it was able to proliferate up to 60 min of treatment (Table 3). Moreover, this strain exhibited a good response in all the bile salt concentrations as high as 1% in all treatment times (Table 5). Considering the relationship to *Bacillus* species and biochemical results, it can be stated that this strain may also have a good effect on our target species.

*Glutamicibacter mysorens* (S_2_I_8_) belong to a group of species that were reclassified from the genus *Arthrobacter* into the newly established genus *Glutamicibacter. *It is a soil bacteria isolated from diverse environments, including mangrove soil and lake sediment [30]*. *It was discovered to produce bioactive compounds with potential pharmacological applications, such as antimicrobial and anticancer peptides [30]. In the present study, the growth response of the strain in pH 3 and 4 was significant while in pH 2, it was able to proliferate for up to 30 min of the treatment period (Table 3). However, this strain exhibited a good response in all the bile salt concentrations as high as 1% in all treatments (Table 5). Given its potential as a source of bioactive compounds and its survival results, it can be considered a potential probiotic candidate in aquaculture for our target species. *Acinetobacter lwoffii *(S_3_I_10_) is the only Gram-negative bacteria isolated from our experimental species. It is an opportunistic pathogen and can are often isolated from soil, water, and healthy or diseased fish [31]. Although healthy fish samples from each station were selected, the appearance of this strain may indicate the degradation of our natural environment. By discarding our waste in the natural environment, we are risking our aquatic organisms continuously. In the present study, it did not show any vitality in any pH (Table 3). Additionally, only a limited response was found in 0.3 and 0.5% bile salt concentrations while in 1% concentration, it could not withstand at all (Table 5).

According to previous reports, one of the most important features of probiotic bacteria is the potential viability at low pH and higher bile salt concentration [5,13,14]. Although, it is generally expected that the bacterial flora from the intestine will exhibit such properties, however, their degree of sensitivity or tolerance varies with strains, age, genetics, environment, and diet [32]. In the present study, the survival and growth of some isolates at low pH indicate they are able to pass through the stomach to the intestine and have higher potentiality as probiotics [5]. While the same isolate(s) exhibited higher bile salt tolerance, it indicated their vigorous activities in the intestine, having the ability to survive under stressful conditions [5,33]. Among the 10 identified bacterial species, most of them are probiotics and they showed a strong impact on different aspects of aquaculture. For application in the culture of stinging catfish, a combination of these probiotic strains can be implemented.

## Conclusion

In this study, some potential probiotic bacteria were isolated from stinging catfish, and the probiotic potentialities of the selected isolates were verified through pH and bile tolerance tests. Based on potentiality tests and previous literature reviews, five isolates such as *B. subtilis*,* S. succinus, M. caseolyticus*,* G. mysorens*, and *B. cereus* were identified as potential probiotic bacteria. The use of species-specific probiotics is considered to perform more effectively and efficiently than unknown sourced commercial probiotics, we believe the findings of this study will be applicable in enhancing the production of stinging catfish in Bangladesh.

## List of Abbreviations

BLAST, Basic local alignment search tool; Bp, Base pair; CFU, Colony forming unit; MRS, De Man–Rogosa–Sharpe; NCBI, National Center for Biotechnology Information; TE buffer, Tris EDTA buffer.
